# Transcription Independent Stimulation of Telomerase Enzymatic Activity by HTLV-I Tax Through Stimulation of IKK

**DOI:** 10.13188/2377-9292.1000024

**Published:** 2021-09-05

**Authors:** M Bellon, Y Yuan, C Nicot

**Affiliations:** 1 Department of Pathology and Laboratory Medicine, University of Kansas Medical Center, USA; 2 Department of Pharmacology, Baylor College of Medicine, USA

**Keywords:** HTLV-1, ATL, leukemia, hTERT, Telomerase, IKK, Tax, HBZ

## Abstract

The persistence and spreading of HTLV-I infected cells relies upon their clonal expansion through cellular replication. The development of adult T cell leukemia (ATLL) occurs decades following primary infection by HTLV-I. Moreover, identical provirus integration sites have been found in samples recovered several years apart from infected individuals. These observations suggest that infected cells persist in the host for an extended period of time. To endure long term proliferation, HTLV-I pre-leukemic cells must acquire critical oncogenic events, two of which are the bypassing of apoptosis and replicative senescence. In the early stages of disease, interleukin-2 (IL-2)/IL-2R signaling likely plays a major role in combination with activation of anti-apoptotic pathways. Avoidance of replicative senescence in HTLV-I infected cells is achieved through reactivation of human telomerase (hTERT). We have previously shown that HTLV-I viral Tax transcriptionally activates the hTERT promoter. In this study we demonstrate that Tax can stimulate hTERT enzymatic activity independently of its transcriptional effects. We further show that this occurs through Tax-mediated NF-KB activating functions. Our results suggest that in ATLL cells acquire Tax-transcriptional and post-transcriptional events to elevate telomerase activity.

## Introduction

### Background

HTLV-I is a human retrovirus associated with the development of an aggressive form of T-cell leukemia known as ATLL [1]. The disease has a dismal prognosis with a survival time of 8-12 months and no cure [2,3]. Hence, the development of novel therapies is greatly needed. HTLV-I provirus replication in ATLL cells *in vivo* occurs mainly through clonal expansion as a result of cellular DNA replication during cell division. Numerous studies have linked disease progression to higher proviral loads [4-6]. The long-term survival and proliferation of HTLV-I-infected T cells correlates with an increased expression of hTERT; and a positive correlation between telomerase activity and the progression of ATLL has been reported [7]. Although the etiologic agent has been well characterized, a mechanistic understanding of the progression of this disease has been elusive. HTLV-I-transformed cells are characterized by the constitutive activation of several cellular signaling pathways such as NF-KB, JAK/STAT, Wnt/β-catenin and PI3K/AKT [8-10]. In addition, studies from several groups have established that the viral oncoprotein Tax plays a central role in the initial steps leading to T-cell immortalization. Tax inactivates multiple cell tumor suppressors and cycle checkpoints, inhibits apoptosis, interferes with DNA repair, and suppresses p53 and Rb, two essential components of the cellular senescence pathway [11]. We have previously demonstrated that hTERT expression is reactivated in HTLV-I transformed cells and HTLV-I Tax stimulates transcription of hTERT mainly through NF-KB and c-Myc [12-15]. In addition, other mechanisms such as IL-2R signaling and activation of the PI3K pathway-may explain high telomerase activity in ATLL cells. In the late stages of ATLL disease, Tax expression gradually decreases and can only be detected in half of acute ATLL patient samples [16-18]. In contrast, expression of the viral HBZ protein appears to be retained in most of the ATLL patient samples analyzed [19,20]. However, it is unclear if and how HBZ contributes to hTERT activity in human T cells and ATLL cells.

Telomeres help to preserve genome integrity and to prevent activation of the senescence program [21-23]. Progressive shortening of the telomeres limits the proliferative capacity of somatic cells [22]. Unprotected short telomeres are recognized and processed as DNA double-strand breaks (DSB) and engage the DNA damage responses (DDR), referred to as telomere dysfunction-induced foci (TIF) [24]. In cells with a functional p53, TIFs induce signals leading to reactivation of p53 transcriptional activities leading to senescence [22]. Initiation of senescence is regulated by the p16INK4a/Rb-dependent pathway and a p53-dependent DDR pathway [25,26]. Most cancer cells avoid senescence by disruption of p53 and p16INK4a and reactivation of hTERT. The ability of telomerase to extend telomere length is subject to complex controls, such as transcriptional and post-transcriptional regulation, and access to the telomeres regulated by components of the shelterin complex [27,28]. Dysfunctional telomeres induced by progressive telomere shortening have been reported to lead to genomic instability, chromosome fusion, aneuploidy, and eventually leading to a pro-cancer genotype[29]. Interbreeding of telomerase-deficient mice leads to critically short telomeres in late generations, which severely affects the onset of tumorigenesis depending on the status of p53 [29]. Studies suggest that dysfunctional telomeres can drive initiation of tumors in the absence of functional apoptosis or senescence checkpoints [30].

We previously demonstrated that Tax can transcriptionally activate the hTERT promoter through multiple pathways. In this study, we investigated the ability of Tax to regulate hTERT enzymatic activity at the post-transcriptional level. Our results suggest that bacterially purified Tax protein can effectively stimulate hTERT activity *in vitro*. Using Tax mutants, we found that NF-KB activating functions of Tax through stimulation of IKK were important for stimulating post-transcriptional activity of hTERT. Our results suggest that HTLV-I has evolved two independent Tax-mediated means to control hTERT expression and enzymatic activity during ATL disease.

### Methods

#### Cell Lines:

HEK and SAOS2 cell lines were maintained in complete Dulbecco modified Eagle’s medium (DMEM) supplemented with 10% fetal bovine serum (FBS) and penicillin/ streptomycin.

#### DNA plasmids:

HTLV-I pcTax, M47 and M22 have been previously described [31]. YFP-hTERT were used, along with Ubc-hTERT vector, that was created by inserting the Ubc promoter into the pHR vector and cloning hTERT cDNA. hTERT-HA-p CIneo was provided by Dr. Weinberg. All vectors were sequenced to verify their integrity. All other plasmids IKK DN, AKT M-, AKT DN and PI3KDN have been previously reported [32-35].

#### Transfection and TRAP Assays:

Telomerase negative HEK or Saos2 cells were transfected with 2μg hTERT (UBc-hTERT or hTERT-HA-pCIneo), and with 2μg Tax or (M22 and M47) and/or 2μg AKT M-, or IKKαDN, IKKβDN, or IKKγDN, using SuperFect transfection reagent (Qiagen), according to the manufacturer’s instructions. 48 hours later, cells were lysed in CHAPs buffer with RNaseOUT (Invitrogen) and 200ng was used for TRAP assays using the TRAPEZE Telomerase Detection Kit (Chemicon International), according to manufacturer’s instructions. TRAP products were run on 8% Tris Boric EDTA gels and stained with SYBR green for visualization.

For luciferase assays, Saos2 cells were transfected with NF-KB luciferase construct (1μg) along with the indicated plasmids (hTERT-3μg, Tax plasmids-1μg, IKK DN plasmids - 2μg). 48hrs post-transfection Saos2 cells were lysed with passive lysis buffer and luciferase activity was measured with Dual Luciferase Reporter Assay System (Promega).

#### Recombinant protein production and GST purification:

Bacterial cells (DH5α) were transformed with pGEX2T vector or pGEX2T-Tax for induction of GST and GST-Tax proteins. Cultures of 500ml with an OD of 0.8 were incubated with IPTG 40 microMolar overnight at room temperature for pGEX2T-Tax and with 1 mM of IPTG for 3 hours at 37C for pGEX2T. Cells were lysed by sonication and bacterial lysates were incubated with glutathione-Sepharose 4B (Pharmacia, Inc.) at 4°C with gentle agitation for 1 hour. The protein-bound Sepharose was washed repeatedly with PBS, and GST fusion proteins were eluted in step fractions containing reduced glutathione and dialyzed overnight against buffer D (20 mM HEPES [pH 7.9], 150 mM KCl, 0.2 mM EDTA, 0.5 mM phenylmethylsulfonyl fluoride, 0.5 mM dithiothreitol, 20% glycerol) at 4°C. Protein fractions were analyzed by SDS-PAGE and stained with Coomasie blue and western blot with a Tax specific antibody. Protein concentration was determined in each fraction and stored at −80°C.

#### Telomeric Repeat Amplification Protocol (TRAP):

To determine telomerase activity, cells were lysed on ice in CHAPS lysis buffer and used in TRAP assays using the Trapeze Telomerase Detection kit (Chemicon) as described by the manufacturer.

## Results

### Post-transcriptional stimulation of telomerase activity by HTLV-I Tax

Although transcriptional activation of hTERT expression is clearly important, studies suggest that transcriptional regulation of hTERT alone is not sufficient to sustain significant telomerase activity in human CD4 T-lymphocytes [36]. In the early phase following infection, the HTLV-I Tax oncoprotein is critical to stimulate T cell proliferation and establish immortalization. In this study, we investigated whether Tax may have additional, transcription-independent, effects on hTERT enzymatic activity. Since we have previously demonstrated that Tax can activate hTERT promoter transcription through multiple pathways including activation of c-Myc, PI3K signaling and inactivation of p53 it is not possible to dissect transcriptional and non-transcriptional effects of Tax in hTERT expressing cells. To alleviate this problem, we used hTERT negative cells. These cells no longer express hTERT due to either hypermethylation of the hTERT promoter or because of activation of the alternative lengthening of telomeres (ALT) pathway.

**Figure 1:**
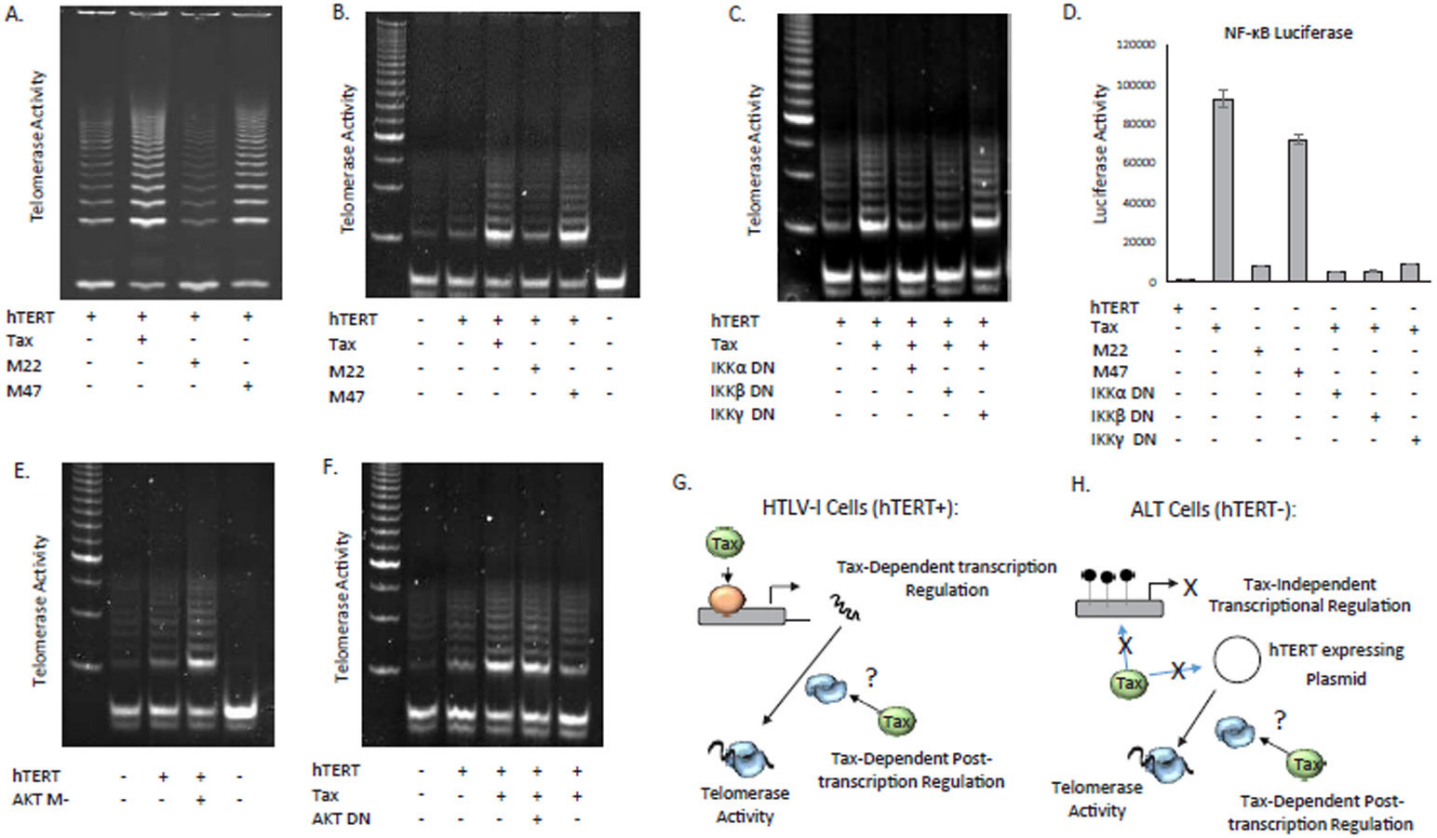
Tax stimulates hTERT activity through the IKK complex. (A-B) HEK (A) or Saos2 (B) cells were transfected with hTERT expression vector in the absence or presence of HTLV-1 Tax wild type or mutant pcTax M47 or pcTax M22. For (A) HEK cells were transfected with UBc driven hTERT expression vector in the absence or presence of HTLV-1 Tax expression vector pcTax. After 48 hours, one half of the cells were lysed in CHAPs buffer and 200ng was used for detection of telomerase activity using TRAP assays. For (B), ALT positive, Saos2, cells were used, and TRAP assays were performed 48 hours after transfection. (C-D) Tax increases telomerase activity through the IKK complex in the ALT cell line, Saos2. (C) Saos2 cells were transfected with an hTERT expression plasmid along with WT Tax, and dominant negative (DN) isoforms of the IKK complex (α,β, and γ); and telomerase activity was measured 48hrs later by TRAP assays. (D) Tax-dependent activation of NF-KB requires IKKα and β. Saos2 cells were transfected with the NF-κB luciferase plasmid along with hTERT, Tax, and/or dominant negative (DN) forms of IKKα, β, or γ. 48 hrs later cells were analyzed for NF-KB luciferase activity. The average of two readings is depicted. (E-F). Tax increases in telomerase activity are partially mediated through the PI3K, but not AKT, pathway activation. (E) Saos2 cells were transfected with an hTERT expression plasmid along with constitutively active AKT (AKT M-); alternatively, Saos2 cells were transfected with hTERT plus WT Tax, and dominant negative (DN) forms of PI3K or AKT (F). Telomerase activity was measured 48hrs later by TRAP assays. Cells transfected with empty vector served as negative controls. (G-H) Schematic representation of the experimental model showing the absence of the transcriptional function of Tax onto the hTERT promoter in telomerase negative ALT cells.

HEK cells which have undetectable levels of endogenous hTERT expression and activity were transfected with a UBc-hTERT expressing vector in the absence or presence of a Tax-expressing vector. After 48 hours telomerase activity was measured using standard TRAP assays, which allow a reproducible and semi-quantitative measurement of telomerase activity. Interestingly, transfection of Tax along with an hTERT expression vector into HEK cells resulted in significantly increased telomerase activity (Figure 1A).

To gain some insights into the mechanism involved, we used established Tax mutants M22 (NF-KB activation defective) or M47 (NF-KB activation active) [31]. Our experiments demonstrated that both the wild type Tax and M47 mutants were able to increase hTERT enzymatic activity while Tax mutant M22 had no significant effects (Figure 1A). These results were also confirmed using another Tax mutant G148V also defective in NF-KB activation (data not shown) [37]. These Tax mutants M22 and G148V are not misfolded and are functional in other known Tax activities such as activation of CREB signaling and inactivation of p53 transcription. We next confirmed these data in another experimental model using human SAOS2 cells. The choice of these cells was prompted by the fact that they do not have any endogenous hTERT expression or telomerase activity and rely on the ALT mechanism for continuous proliferation [38]. Again, our results demonstrated that Tax could stimulate telomerase activity in a transcription-independent manner that requires the NF-KB activity of Tax (Figure 1B).

**Figure 2:**
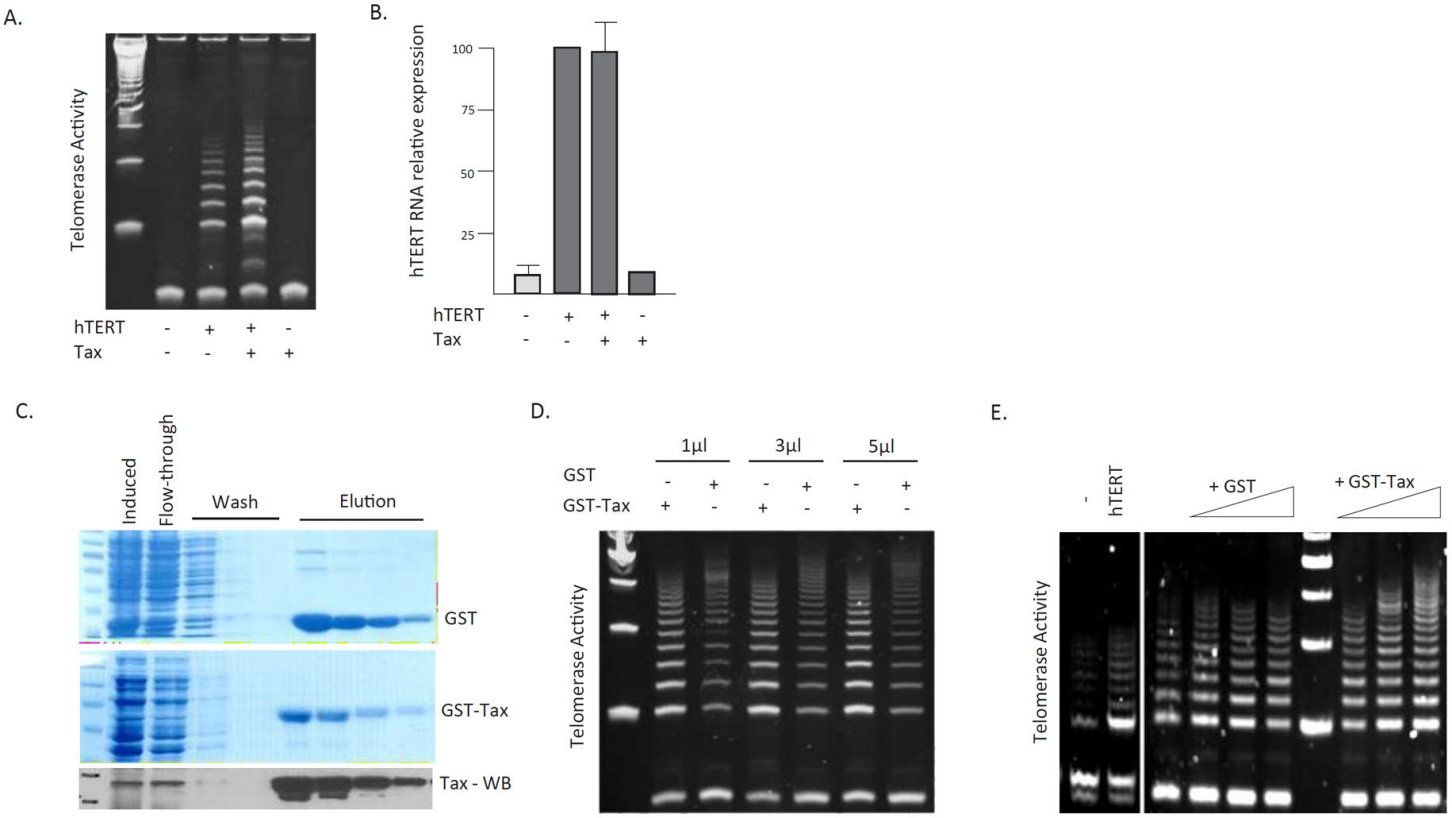
Post-transcriptional activation of hTERT by HTLV-1 Tax. (A) HEK cells were transfected with UBc driven hTERT expression vector in the absence or presence of HTLV-1 Tax expression vector pcTax. After 48 hours telomerase activity was measured using TRAP assays. (B) Half of the cells from (A) were used to extract total RNA and real-time quantitative PCR was performed to detect expression of hTERT in the absence or the presence of Tax. (C) GST and GST-Tax fusion proteins were purified from DH5α bacteria cells transformed with pGEX2T and pGEX2T-Tax following 40μM IPTG induction overnight. Coomassie stained SDS page gels are presented showing the purification and Western blot confirmation of GST-Tax using a Tax mouse monoclonal specific antibody. (D-E) *In vitro* telomerase activity by Tax. Cellular extracts from HEK (D) or Saos2 (E) cells transfected with hTERT expression vector were mixed with equal amounts of GST or GST-Tax recombinant protein and incubated at 37C for 30 minutes then assayed for telomerase activity. (D) Telomerase activity was measured after incubation with 1 μl, 3 μls, or 5μls of GST or GST-Tax purified protein.

HTLV-I Tax has been shown to stimulate the activity of the IKK complex. Since Tax-mediated activation of NF-kB stimulated telomerase activity independently of any transcriptional effect we hypothesized that activation of the IKK complex by Tax resulted in increased hTERT activity. To our knowledge there are no reports showing that components of the IKB kinase (IKK) complex can stimulate telomerase activity in a transcription-independent manner. Since Tax-mediated NF-KB activation M47 mutant, and not M22, was able to stimulate telomerase activity, we next used dominant negative (DN) mutants of the different subunits of the IKK complex [32,33]. HTLV-I Tax has been shown to stimulate IKKα and IKKβ kinase activities and to interact with IKKγ, a modulator of the complex devoid of any kinase activity. As expected from our results presented in Figure 1, SAOS2 cells transfected with hTERT and Tax presented higher telomerase activity than cells transfected with hTERT vector alone (Figure 1C). Both IKKα DN and IKKβ DN but not IKKγ DN, were able to abolish Tax stimulation of telomerase activity in SAOS2 cells as shown by a return to levels seen in the hTERT vector expression alone (Figure 1C). The ability of these mutants to function properly as dominant negative mutants was further demonstrated in luciferase reporter assays (Figure 1D). As expected, all three dominant negative vectors were able to efficiently suppress Tax-mediated activation of the NF-kB luciferase reporter (Figure 2D).

AKT has previously been shown to phosphorylate hTERT and thereby to increase its enzymatic activity [34]. To confirm these observations in our experimental system, we next used a constitutively active form of AKT (AKT M-), mutated on its myristylation site [35]. Transfection of AKT M- into SAOS2 cells, indeed increased telomerase activity (Figure 1E). In addition to AKT, we have previously reported that the PI3K pathway can also stimulate telomerase activity in Tax expressing cells [39]. To identify if either AKT or PI3K was important in Tax-mediated stimulation of telomerase activity we transfected dominant negative mutants AKT DN or PI3K DN [35,40], along with Tax into SAOS2 cells. Our experiments indicated that Tax activity was not mediated through AKT as no change in telomerase activity was detected (Figure 1F). In contrast, a partial reduction in telomerase activity, albeit not to original levels seen without Tax, was seen in the presence of PI3K DN expressing vector (Figure 1F). Although PI3K is known to activate AKT and AKT can phosphorylate IKK, our results suggest that Tax activation of the IKK complex stimulates post-transcriptional telomerase activity independently from AKT. The results in Figure 1 are summarized in 1G and 1H. Since it is established that Tax can activate hTERT promoter transcription through multiple pathways it is not possible to distinguish transcriptional and non-transcriptional effects of Tax in hTERT (positive) expressing cells (Figure 1G). Therefore, we used hTERT negative cells that no longer express hTERT, to examine only the post-transcriptional Tax effects on telomerase (Figure 1H). With the use of these cells, hTERT expression plasmids can be added to provide telomerase activity only through the stimulation of the hTERT, plasmid, promoter. In our model system, Tax will have no transcriptional effect on either the hTERT promoter in ALT cells or on the transfected hTERT expression vector. Therefore, any activation of telomerase activity is attributed to post-transcriptional effects of the transfected Tax protein.

Several hypotheses may explain how Tax expression may affect telomerase activity in a transcription independent manner. Tax may alter the sub-cellular localization of hTERT, Tax may alter transcription factors influencing hTERT expression, or Tax may deregulate signal transduction pathways directly, independently of Tax transcriptional activities, to increase telomerase activity. We, again, transfected HEK cells with a plasmid expression vector driving hTERT expression and confirmed post-transcriptional activation of telomerase activity in the presence of Tax (Figure 2A). We then took RNA from the same cells and analyzed transcriptional activation of the hTERT promoter. This was not the result of an increase in mRNA expression from the endogenous or the UBc-driven transfected hTERT vector since similar levels of hTERT mRNA expression were detected by real time quantitative RT-PCR (Figure 2B). Together these results suggested a novel role of Tax in stimulation of hTERT telomerase activity without increasing hTERT mRNA expression level. These results confirm that Tax activates hTERT post-transcriptionally.

Previous studies have shown that bacterially purified GST-Tax is still able to activate cellular kinases, including IKK, *in vitro* [41]. To differentiate between the different hypotheses described above, we set up an *in vitro* assay so that hTERT subcellular localization and *de novo* gene transcription would no longer be influential factors. Tax cDNA was cloned into the pGEX-2T for expression in E. coli DH5α cells and purified (Figure 2C). Protein extracts, from HEK cells (Figure 2D) or Saos2 cells (Figure 2E) transfected with an hTERT expression vector or a control vector, were quantified and an equal amount was mixed with increasing amounts of bacterially purified GST-Tax or GST protein control. The mixture was incubated at 37C and telomerase activity was measured by TRAP assay. Our results showed that bacterially purified GST-Tax is able to stimulate hTERT activity in a dose-dependent manner while GST control had no effect (Figure 2B and 2C).

Altogether these results unequivocally demonstrate that Tax protein can increase hTERT enzymatic activity in a transcription-independent manner.

## Discussion

In this study, we demonstrate for the first time that the HTLV-I oncogenic Tax protein can stimulate human telomerase enzymatic activity independently of any transcriptional activities. Initial studies were performed by transfecting HEK and SAOS2 cells in which no hTERT promoter expression can be detected. These data were then confirmed using bacterially purified Tax protein and *in vitro* assays to measure telomerase TRAP assays. Telomerase activity can increase as a result of higher hTERT mRNA expression, sub-cellular localization, or phosphorylation. Our results suggest that Tax can activate signaling pathways resulting in increased phosphorylation and hTERT activity. A better understanding of this mechanism may offer new ways to interfere with hTERT activity in ATLL cells. This may be important since we have previously shown that blocking hTERT activity triggers senescence of ATLL cells carrying a wild type p53 gene [12]. The fact that Tax activates hTERT enzymatic activity in a transcription independent manner may have important consequences for HTLV-I transformed cells. Most ATL cells express very low levels of Tax protein during the S phase of the cell cycle. Hence, timely stimulation of telomerase activity may allow extension of telomere ends during DNA replication when transcription is not available.

We also utilized different dominant negative mutants to specifically block Tax-mediated activation of IKK, AKT or PI3K. Results from these studies demonstrate that Tax can stimulate telomerase activity though activation of IKK and/or PI3K signaling pathways. Although it is generally accepted that PI3K can activate downstream of IKK through AKT, our results suggest that either PI3K can directly stimulate telomerase activity or enhance IKK activity in an AKT-independent manner in the presence of Tax. Tax has been shown to interact with and activate NF-KB inducing kinase (NIK) to phosphorylate and activate IKKα and IKKβ [42]. We think that the PI3K DN partial rescue may result from protein kinase C (PKC) signaling since previous studies have linked PKC and hTERT activity. In addition, signaling through PI3K can activate mitogen-activated protein kinase 8 (MAP3K8), which in turn can activate IKKα and IKKβ or phosphorylate NIK [43].

We previously reported immortalization of human primary T cells with a lentiviral Tax only expression vector [44]. In these cells, both hTERT mRNA and telomerase activity are readily detected and required for persistent proliferation. However, several studies have suggested that during ATL disease progression Tax expression is progressively lost and replaced by HBZ expression. HBZ mRNA encoded from the 3’LTR is partly complementary to the Tax mRNA sequence and can potentially act as anti-sense RNA. In HTLV-I-infected cells it is believed that silencing of Tax expression progressively results in viral LTR promoter shut-down, thereby allowing HBZ expression from the 3’ LTR in the absence of Tax [20]. Despite this, studies using de-methylating agents have shown that reactivation of Tax expression can be detected in ATLL cells. In addition, active replication as obtained by culturing ATLL patient cells *ex vivo*, leads to rapid Tax re-expression. Recent studies have also suggested cell cycle dependent expression of Tax at “under the radar” levels in most ATLL cells [45]. These observations are consistent with the high level of CTL directed against Tax observed in HTLV-I symptomatic patients. While not addressed in this study, preliminary data from our lab demonstrates that HBZ is able to stimulate telomerase activity in T-cells. It is possible that HBZ itself may take an active role in controlling Tax expression possibly through antisense RNA duplex formation. It is possible that early Tax expression drives both transcriptional and post-transcriptional activation of telomerase activity and that later, there is a shift to HBZ-mediated telomerase activation to prevent replicative senescence of the leukemic cell.

## Conclusion

Our results demonstrate that HTLV-I Tax oncoprotein can post-transcriptionally stimulate telomerase enzymatic activity and may serve as therapeutic target in early stages of ATL disease.
